# Presence of Methicillin-Resistant *Staphylococcus aureus* in Pigs in Peru

**DOI:** 10.1371/journal.pone.0028529

**Published:** 2011-12-08

**Authors:** Carmen S. Arriola, Mariella E. Güere, Jesper Larsen, Robert L. Skov, Robert H. Gilman, Armando E. Gonzalez, Ellen K. Silbergeld

**Affiliations:** 1 School of Veterinary Medicine, San Marcos Major National University, Lima, Peru; 2 Department of International Health, Johns Hopkins Bloomberg School of Public Health, Johns Hopkins University, Baltimore, Maryland, United States of America; 3 Department of Microbiological Surveillance and Research, Statens Serum Institut, Copenhagen, Denmark; 4 Department of Environmental Health Sciences, Johns Hopkins Bloomberg School of Public Health, Johns Hopkins University, Baltimore, Maryland, United States of America; Montana State University, United States of America

## Abstract

We report the first detection of methicillin-resistant *Staphylococcus aureus* isolates in pigs in Peru. The isolates belong to a livestock-associated lineage previously reported in North America and Europe, CC398, and a highly virulent USA300-like ST8-IV variant, which is the predominant community-associated lineage in Latin America.

## Introduction

Recent reports of methicillin-resistant *Staphylococcus aureus* (MRSA) in livestock, particularly pigs, and in individuals with contact to livestock provided the first evidence of the existence of a true livestock-associated MRSA (LA-MRSA) reservoir throughout Europe [1], [2]. Subsequently, LA-MRSA has been identified in Canada [3] and in the United States [4]. LA-MRSA isolates have important genotypic and phenotypic characteristics (reviewed in [5]): (1) they are nontypeable by pulsed-field gel electrophoresis (PFGE) using *Sma*I [6]; (2) many of them belong to clonal complex (CC)398 by multi-locus sequence typing (MLST); (3) the staphylococcal chromosomal cassette (SCC)*mec* elements encoding β-lactam resistance are different from those carried by other MRSA genotypes commonly found in the community and healthcare settings [7]; (4) they often exhibit co-resistance to many non-β-lactam antimicrobials (e.g., antibiotics and metals) including those commonly used in animal production [7], [8]; and (5) the majority of isolates lacks toxins such as Panton-Valentine leukocidin (PVL) and other enterotoxins [8], [9].

With a population of about 80 million pigs (http://faostat.fao.org), Latin America has a large potential reservoir for LA-MRSA. In Peru, a variety of different pig production systems exists, including scavenging pigs, semi-intensive backyard production, small-scale intensive production, and large-scale confined production, which are characterized by specific management practices and biosecurity risks. We undertook this study to investigate whether MRSA is present in different production systems in Peru.

## Methods

From February through November 2009, nasal swabs were taken from a total of 240 ready-to-market pigs (143–150 days old), including 20 pigs from each of 6 large-scale confined production holdings located in agricultural areas of the Department of Lima and 20 scavenging pigs from each of 6 rural communities in the Department of Tumbes. The Johns Hopkins University Animal Care and Use (ACUC) Committee approved the research protocol (SW08H111). The different pig production systems were categorized based on the definitions of the Food and Agriculture Organization of the United Nations [10]. Samples were stored in Stuart transport medium (Eurotubo, Deltalab, Barcelona, Spain) and streaked on mannitol salt agar (MSA, Merck, Darmstadt, Germany) supplemented with cefoxitin (4 µg/ml) within 24 h. Presumptive MRSA isolates were subcultured after 24 h onto oxacillin resistance screening agar base (ORSAB, Oxoid, Basingstoke, United Kingdom) and identified by coagulase test and multiplex PCR for 16S rRNA, *mecA,* and *nuc* genes as described elsewhere [11]. All MRSA isolates were characterized by staphylococcal protein A *(spa)* typing using the Ridom Staph Type standard protocol (http://www.ridom.com) and the Ridom SpaServer (http://spa.ridom.de/index.shtml) and were typed for SCC*mec* using a multiplex PCR assay described by Kondo et al. [12].

## Results

Eight (40%) of 20 pigs from 1 of 6 sampled large-scale holdings carried MRSA; all isolates belonged to *spa* type t571 and carried SCC*mec* type V. In comparison, only 1 (5%) of 20 scavenging pigs from 1 of 6 sampled communities carried MRSA; this isolate belonged to *spa* type t008 and carried SCC*mec* type IVc.

A representative t571 isolate and the single t008 isolate were selected for further analyses. MLST types were determined (http://www.mlst.net), and the presence of 6 virulence-associated genes (*lukF-PV*, *lukF-PV*, *arcA*, *sek*, *seq*, and *bsaA*) was investigated, using PCR assays and primers that have been described elsewhere [13]. The USA300-0114 strain was used as a positive control for the PCR amplifications. The antimicrobial susceptibility profiles (spectinomycin, gentamicin, kanamycin, tobramycin, rifampin, trimethoprim-sulfamethoxazole, clindamycin, erythromycin, linezolid, chloramphenicol, mupirocin, ciprofloxacin, minocycline, tetracycline, and fusidic acid) were determined by the agar dilution method using Neo-Sensitabs (Rosco, Taastrup, Denmark), in accordance with the Clinical Laboratory Standards Institute guidelines [14]. Screening for reduced susceptibility to glycopeptides was performed on brain-heart infusion agar supplemented with 5 µg/ml teicoplanin followed by testing of positive MRSA isolates using Etest glycopeptide-resistance detection strips (vancomycin, 32–0.5 µg/ml; teicoplanin, 32–0.5 µg/ml) (bioMérieux, Marcy l'Etoil, France) as described by Fitzgibbon et al. [15].

The t571 isolate had genotypic and phenotypic characteristics of LA-MRSA: it belonged to ST398, was resistant to 6 of 16 non-β-lactam antibiotics (clindamycin, erythromycin, chloramphenicol, ciprofloxacin, minocycline, and tetracycline), and lacked all investigated virulence-associated genes. In contrast, the t008 isolate showed genotypic and phenotypic characteristics related to a USA300-like ST8-IV variant, which is the predominant community-associated MRSA (CA-MRSA) lineage in Latin America [13], [16], [17]; it belonged to ST8, was susceptible to all 16 non-β-lactam antibiotics, and carried *lukF-lukS* genes encoding PVL as well as genes encoding enterotoxins (*sek* and *seq*) and bacteriocin (*bsaA*) but lacked *arcA* (an indicator of the arginine catabolic mobile element).

## Discussion

This study provides the first evidence of a porcine reservoir of LA-MRSA-ST398-V in Latin America and the presence of a highly virulent USA300-like CA-MRSA lineage, ST8-IV, in Peru. However, the extent of the MRSA reservoir in animals remains speculative because of limitations in sample size and sampling locations.

The origin of LA-MRSA-ST398-V in Latin America is not known. Methicillin-susceptible *S. aureus* (MSSA) ST398 strains characterized by *spa* type t571 and lack of the *lukF*-*lukS* genes encoding PVL have been reported in non-hospitalized and hospitalized community-dwelling individuals with no livestock-associated risk factors in Latin America [18], [19], [20], which may suggest that LA-MRSA-ST398-V is a derivative of a progenitor CA-like MSSA ST398 strain. On the other hand, the emergence of LA-MRSA in Latin America could also be due to animal importation from other countries with established LA-MRSA reservoirs, as previously reported in Europe [21], but not yet extensively studied in Peru.

While high rates of MRSA isolation have been reported among hospitalized patients in the Andean region of Latin America [13], LA-MRSA carriage and disease in individuals have not been reported. However, we hypothesize that the proportion of LA-MRSA in humans will be higher in areas where LA-MRSA has been successfully established in the pig population, as previously reported in The Netherlands [22].

Of note, a single MRSA isolate from a scavenging pig was determined to be of the highly virulent USA300-like ST8-IV lineage, which is the predominant and almost exclusive CA-MRSA clone in the Andean region of South America, including Peru [13], [16], [17]. Such scavenging pig production systems are characterized by little confinement of the animals or lack thereof, such that pigs have access to human feces and greater likelihood of exposure to human strains of MRSA. Consequently, our finding is likely to reflect infrequent human-to-animal transmission, rather than the existence of a true animal reservoir.

In conclusion, our findings demonstrate that LA-MRSA is present in large-scale pig production systems in Peru. However, the extent of the porcine MRSA reservoir and their implications for MRSA colonization and disease in humans in Peru and other Latin American countries have still to be determined.
